# A Misdiagnosed Familiar Brooke–Spiegler Syndrome: Case Report and Review of the Literature

**DOI:** 10.3390/jcm13082240

**Published:** 2024-04-12

**Authors:** Tito Brambullo, Alberto De Lazzari, Arianna Franchi, Eva Trevisson, Maria Luisa Garau, Federico Scarmozzino, Vincenzo Vindigni, Franco Bassetto

**Affiliations:** 1 Clinic of Plastic Surgery, Neurosciences Department, University of Padua, 35131 Padua, Italy; tito.brambullo@aopd.veneto.it (T.B.); alberto.delazzari@aopd.veneto.it (A.D.L.); arianna.franchi@aopd.veneto.it (A.F.); vincenzo.vindigni@unipd.it (V.V.); 2 Clinical Genetics Unit, Department of Women and Children’s Health, University of Padova, 35131 Padua, Italy; eva.trevisson@unipd.it (E.T.); marialuisa.garau@aopd.veneto.it (M.L.G.); 3 Surgical Pathology & Cytopathology Unit, Department of Medicine (DIMED), University of Padova, 35131 Padova, Italy; federico.scarmozzino@aopd.veneto.it

**Keywords:** adnexal neoplasm, Brooke–Spiegler syndrome, cylindroma, eccrine cylindromatosis, neurofibromatosis type 1, trichoepithelioma, spiradenoma, spiradenocylindroma

## Abstract

Aim of the report: Brooke–Spiegler syndrome (BSS) is a rare autosomal dominant disease characterized by the growth of cylindromas, spiradenomas, trichoepitheliomas, or their combination. These neoplasms usually begin in the second decade and progressively increase in number and size over the years. Diagnosis necessitates consideration of family history, clinical examination, histological findings, and genetic analysis. The aim of this paper is to explore the clinical overlap between Brooke–Spiegler syndrome (BSS) and neurofibromatosis type 1 (NF1). We aim to highlight the challenges associated with their differential diagnosis and emphasize the lack of standardized diagnostic criteria and treatment approaches. Case presentation: Hereby, we introduce the case of a 28-year-old male referred for suspicion of neurofibromatosis type 1 (NF1) who initially declined the recommended surgical excision for a scalp mass. After four years, he returned with larger masses of the scalp, and underwent excision of multiple masses, revealing cylindromas, spiradenomas, and spiradenocylindromas. Family history reported similar tumors in his father, who was also diagnosed with NF1 for the presence of multiple subcutaneous lesions on the scalp. Clinical overlap led to a genetic consultation, but testing for CYLD mutations yielded no significant variations. Despite this, the strong family history and consistent findings led to a revised diagnosis of Brooke–Spiegler syndrome, correcting the initial misdiagnosis of NF1 syndrome. Conclusions: Thanks to the evolving landscape of BSS research over the past two decades, its molecular underpinnings, clinical presentation, and histopathological features are now clearer. However, a thorough family history assessment is mandatory when BSS is suspected. It is our belief that a multidisciplinary approach and cooperation between specialists are essential when dealing with BSS. By sharing this case, we hope to underscore the importance of considering BSS as a differential diagnosis, especially in cases with atypical presentations or overlapping features with other syndromes like NF1.

## 1. Introduction

Brooke–Spiegler syndrome (BSS) is a rare autosomal dominant disorder characterized by the appearance of adnexal tumors, usually on the scalp, face, and occasionally on the trunk [1]. The estimated incidence of Brooke–Spiegler syndrome (BSS) is approximately 1 in 100,000 in the UK [2]. Over the past 20 years, studies analyzing the total number of patients affected by BSS have encompassed around 150 individuals. These findings predominantly stem from two systematic reviews and two randomized controlled trials. Other studies primarily comprise case reports, contributing only a small number of patients to the overall analysis.

The disease is more common among women and usually occurs during the second or third decade of life, with the tumors growing in both size and number over time [3]. Usually, nodules are typically small (0.5–3 cm), but there are also larger lesions that can achieve up to 7 cm in diameter, resulting in major aesthetic concerns for the patient. The number of lesions can vary from patient to patient; some may develop only one to a few, while others may exhibit the emergence of hundreds of nodules all over the body. Although malignant transformation is rare, it should be considered in cases of rapid enlargement and bleeding [3].

Clinically, BSS is characterized by the development of cylindromas, trichoepitheliomas, spiradenomas, and spiradenocylindromas on the skin. Trichoepitheliomas (Multiple Familial Trichoepitheliomas, or MTFs) are the only manifestation for some patients, while others have cylindromas (familial cylindromatosis). Despite their historical distinction as different nosological conditions, these syndromes are now recognized as variations of BSS because of potential clinical and histological overlap under the definition of CYLD cutaneous syndrome [2,4].

The oncosuppressor CYLD is the only gene that contributes to the development of BSS. However, to accurately diagnose this condition, it is necessary to take into account family history, clinical examination, histological findings, and genetic analysis. In fact, a CYLD gene mutation cannot be detected in up to 20% of BSS patients [5].

A role for alterations of the oncosuppressor gene PTCH1 on chromosome 9q22.3 in promoting BSS was hypothesized by early studies that detected pathogenic variations of this gene in patients affected by trichoblastomas and trichoepitheliomas [6]; however, subsequent works did not confirm this finding [7]. PTCH1 is one of the causative genes of nevoid basal cell carcinoma syndrome [8], and it is found mutated in various sporadic tumors [9].

Beyond these findings, a small percentage of patients seem to develop Brooke–Spiegler syndrome even in the absence of an ascertainable oncosuppressor gene alteration.

In this subgroup of patients, the differential clinical diagnosis with neurofibromatosis type 1 (NF1) could be arduous due to the considerable overlap between the two syndromes.

Both conditions are characterized by the development of indolent skin lesions, which may slowly grow during a long time lapse, without causing any other significant symptoms. The malignant transformation is rare, but possible in each of the two, and, usually, the excision strategy is to biopsy selected lesions that evolve rapidly or cause aesthetic discomfort. Rarely, a complete eradication of all appreciable lesions is requested by the patient, due to unavoidable tissue scarring impairment.

Surgical excision, electrocautery, and laser ablation (e.g., CO_2_ or YAG laser) are all possible therapeutic interventions, but it should be taken into account that only the first procedure allows for histologic examination of the specimen, and, thus, a definitive assessment of the malignant potential. A clinical follow-up is commonly recommended for the aforementioned reasons [10].

This paper describes a misdiagnosed case of Brooke–Spiegler syndrome, which was initially treated as NF1. It intends to highlight the potential clinical overlap between these syndromes and emphasizes the necessity of a multidisciplinary approach when dealing with rare diseases.

## 2. Review of the Literature

### 2.1. Database Research

In order to compile a review of the literature regarding Brooke–Spiegler syndrome, a literature search was performed on the PubMed^®^ database using the following research criteria: “Brooke–Spiegler Syndrome and Neurofibromatosis type 1”, “BSS and NF1”. Only articles written in English were considered.

In the last twenty years, only 254 articles have been published in the PubMed^®^ database on Brooke–Spiegler syndrome (BSS). Of these, only three are classified as levels 1–2 of evidence. In two randomized controlled trials (RCTs), only CYLD-mutated patients were investigated [2,11]. In one systematic review [12], patients were not genetically tested. Two other studies can be classified as level 3 (clinical trials), of which one focuses on the skin lesions’ response to the topical use of salicylic acid in mutated patients [13], and only one article reported two cases of CYLD and PTCH1 mutation-negative patients [14]. In the latter, the molecular analysis of the coding regions flanking the exons of the CYLD and PTCH1 genes did not detect any germline or somatic mutations. The authors concluded that in CYLD and PTCH1 mutation-negative patients with Brooke–Spiegler syndrome, other genes might be mutated. However, this may depend on the incomplete sensitivity of the molecular techniques employed, or on the presence of mosaics. Interestingly, large deletions that are usually missed by traditional sequencing methods have rarely been reported in BSS [15]; in addition, deep intronic changes, or variations in the promoter region, are usually not covered by standard analyses [16,17].

Twenty-six non-systematic reviews were identified in the research, along with numerous other studies (observational, case reports) that had lower amounts of evidence. The concrete possibility of analyzing this syndrome is impacted by multiple biases, including the wide allele heterogeneity, large rearrangements and deep intronic variants, and the presence of mosaicism [18]. In addition, there is great variability in the affected population, from those with a few well-defined subcutaneous nodules to those with a multitude of small lesions differing in size and appearance. Thirdly, a systematic registry of patients affected by the condition is lacking due to the ambiguity of its definition, which has interfered with the data record so far.

### 2.2. The Diagnostic Challenge

The difficulty in making an early diagnosis, as shown in our case, may be the main factor that leads to unsuccessful treatment. With this focus, Kazakov et al. [4] noted an evolution in the definition of this syndrome over the years, recognizing that familial cylindromatosis and multiple familial trichoepithelioma may converge under the umbrella of BSS. In contrast, certain authors tend to label this condition as CYLD cutaneous syndrome (CSS) based on the key role of the CYLD gene on chromosome 16q12-q13 in disease development. Kazakov and colleagues [6] investigated a small population of affected patients, confirming that MFT is likely a phenotypic variant of BSS, and reported a germline mutation rate in CYLD of approximately 40%. The authors suggested that in CYLD mutation-negative cases, another gene may be involved, but failed to underline any role of a mutated PTCH1 gene. In the past, it was suggested that PTCH mutation was implicated in the etiopathogenesis of BSS based on occasional findings [5,19].

However, the lack of a molecular diagnosis impedes the confirmation of the clinical diagnosis of BSS, which may be, nevertheless, established by integrating histopathology findings with personal and family histories [1].

The histological characteristics of these tumors are similar, according to Kazakov et al., who note that spiradenomas are comprised of nodules that contain basaloid cells arranged in trabecular, reticular, or solid patterns, along with pale cells and lymphocytes. Cylindromas are formed by monomorphic basaloid cell nodules that contain eosinophilic material, and have a small number of lymphocytes present. Spiradenocylindromas are characterized by a combination of both histological findings [20].

According to Dubois et al. [21], there are no uniform diagnostic criteria that have been established yet. It is suggested by some authors to suspect BSS when one or more cylindromas, spiradenomas, or spiradenocylindromas on the face or scalp develop in the second–third decade and then increase in number and size. Likewise, BSS should be considered for any instances where cylindroma or spiradenoma is found by chance while patients are undergoing imaging studies or if salivary gland neoplasms are present in individuals with cylindroma, spiradenoma, or trichoepithelioma. In the event of a suspected case, it may be crucial to conduct a family history investigation, as first-degree relatives are usually affected [4].

Some authors suggest investigating CYLD variants when two biopsies on the same patient confirm the presence of these neoplasms, or if a single biopsy shows a positive result, if a first-degree relative has removed one of these neoplasms [22]. Arefi et al. [23] suggest that mosaicism should be considered, as patients may have clinical and histological features similar to BSS in the absence of family history, but sequencing analysis of CYLD may not reveal genetic variations. To avoid this inconvenience, mutation research, not on blood tests, but on histological specimens is warranted.

## 3. Case Report

**Figure 1:**
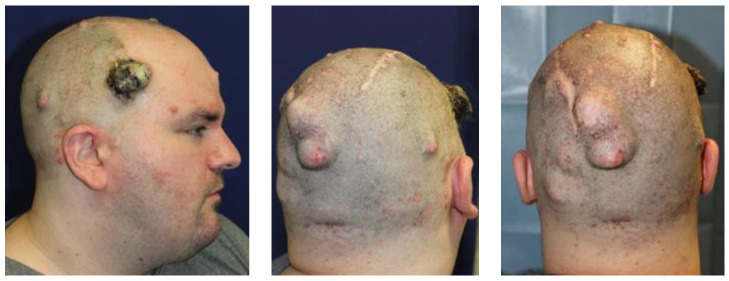
The enlarging mass on the right temple and the other tumors.

A 28-year-old male with a clinical diagnosis of neurofibromatosis type 1 (NF1) presented to our clinic in 2018 with a progressively enlarging scalp mass [24]. The mass appeared as an exophytic red papule, without ulceration, and the patient reported increasing discomfort associated with the tumor. The family history was significant, as his father, paternal aunt, and grandmother all presented a similar clinical picture and were diagnosed with NF1 [25]. A wide surgical excision and reconstruction with local flaps were recommended, but the patient declined the surgery and discontinued follow-up for four years. In March 2022, the patient returned to our clinic due to further enlargement of the mass, which had reached approximately 10 × 8 cm^2^ (Figure 1). 

**Figure 2:**
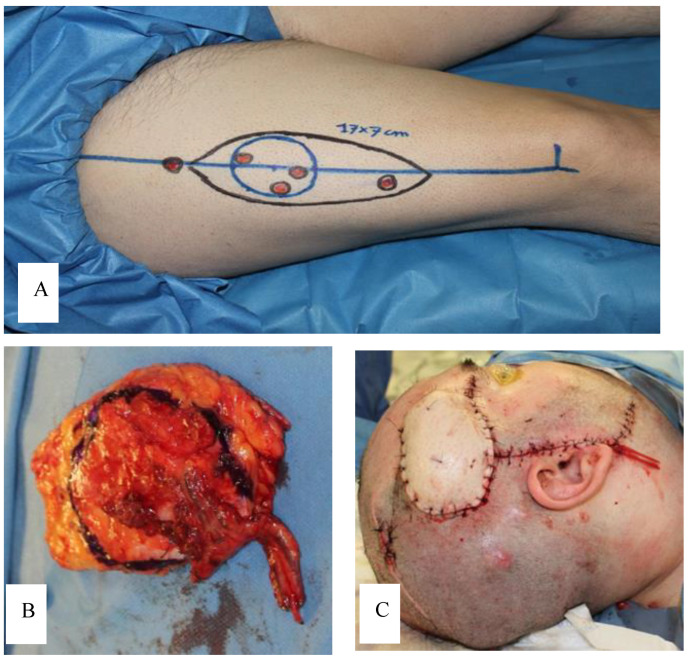
(**A**) Surgical planning; (**B**) ALT flap; (**C**) final result.

Under general anesthesia, a wide excision of the masses located on the right temple, right parietal region, vertex of the scalp, occipital region, and frontal area was performed. Reconstruction was carried out using an anterolateral thigh (ALT) free flap (Figure 2). To achieve disease-free margins, we chose the subperiosteal plane as our preferred plane. Since microsurgery was involved, and considering the dissection plane and the duration of the surgery (approximately six hours), general anesthesia was mandatory.

The healing process was uneventful; the patient was discharged after five days post-surgery and had a complete healing of the wounds within three weeks. The patient is already scheduled for further excision of the other scalp lesions.

**Figure 3:**
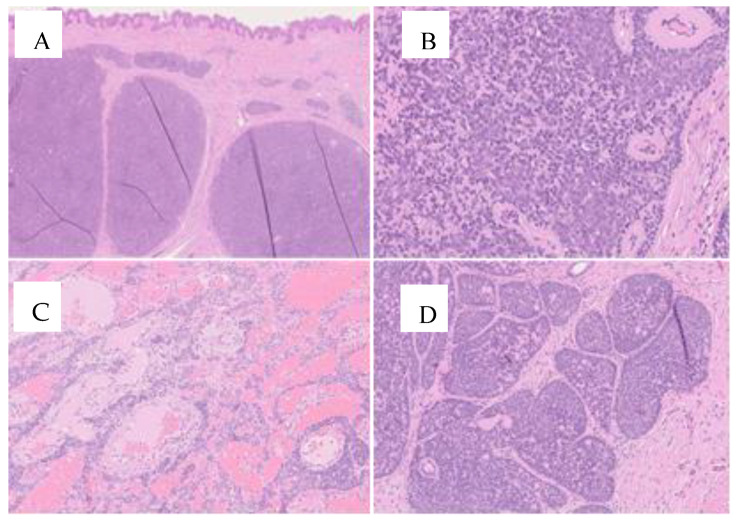
On microscopic examination, we note in Figures (**A**–**C**), a spiradenoma, which consists of multiple dermal nodules (**A**) surrounded by a thick basement membrane and composed of basaloid cells with minimal mitotic activity. Basement membrane material was also detected inside the neoplastic nests in the form of round deposits (**B**). The stroma between the tumor aggregates showed edema and numerous dilated vessels (**C**). In Figure (**D**), the presence of cylindromatous areas characterized by a “jigsaw puzzle” arrangement of the neoplastic nodules prompts the diagnosis of cylindroma. (H&E stain; original magnification 1.25×, 10× and 20×).

The histological examination diagnosed the presence of cylindromas for the right parietal mass, spiradenomas for the ulcerated mass in the right temporal region, for the mass on the vertex of the scalp, and for the frontal neoformation; spiradenocylindroma was the diagnosis for the occipital mass. The comprehensive clinical, anamnestic, and morphological findings suggested investigating the possibility of Brooke–Spiegler syndrome (Figure 3).

Upon reviewing his family history, it was noted that his father had undergone several excisions of tumors on his scalp, face, and back. Interestingly, these tumors were not identified as neurofibromas but rather as a combination of cylindromas, spiradenomas, and spiradenocylindromas. One of these tumors was a combination of spiradenocarcinoma and cylindroma [26].

A multidisciplinary discussion involving our pathologists was conducted, revealing a complete overlap in histological findings between the father’s and son’s specimens. Subsequently, a genetic consultation was initiated for the son, as his father had passed away in 2018 from colorectal and pancreatic carcinomas. Sequencing of the oncosuppressor gene CYLD was performed, but no significant variations were detected. However, considering the compelling family history and consistent clinical and histological findings, the diagnosis was revised to Brooke–Spiegler syndrome, correcting the initial misdiagnosis of NF1 syndrome [27,28].

A new aspect of this diagnostic challenge emerged with the correlation to the clinical history of the patient’s father, who, ten years prior, was himself a patient at our institute for similar reasons (multiple spiradenomas and cylindromas of the scalp and trunk). The known family history and histological specimens led us to this diagnosis.

We firmly believe that a thorough investigation of familial history must be conducted whenever BSS is suspected due to its clinical and genetic heterogeneity. This case highlights the need for a more meticulous multidisciplinary evaluation for future patients.

## 4. Discussion

Laser ablation or electrocautery can be used to remove cutaneous or subcutaneous neurofibromas, just like BSS lesions. Malignant transformation is a potential outcome of neurofibromas and other NF1-related tumors, and may necessitate radical surgical excision and/or chemotherapy [29].

BSS and NF1 are both rare conditions and are characterized by wide clinical variability that causes many patients to remain undiagnosed for years or even generations [30].

Our report describes an adult patient with BSS who was clinically misdiagnosed and misinterpreted as NF-1.

This clinical issue needs to be more focused on because this is not the first time that such a misunderstanding has been reported [31]. Thus, an accurate personal and familiar assessment, as well as an appropriate diagnostic algorithm, appear to be of major importance when evaluating patients with multiple long-lasting cutaneous lesions. An autosomal dominant pattern of inheritance, tumor development at an early age, and multiple lesions are common features described for genodermatoses, such as BSS and NF-1. Other clinical entities, such as autosomal dominant familial angiolipomatosis, have also been mistaken for NF-1 because of similar clinical features in the literature [32]. Inappropriate specific knowledge and insufficient information, like the absence of careful microscopic examination, have been described as possible causes of these misinterpretations [33].

The initiative of the fifth edition of the WHO classification of the Head and Neck Tumours established a new section dedicated to familial/heritable tumor syndromes [34]. BSS was included along with other syndromes that result in the growth of tumors and lesions in the head and neck area, emphasizing the necessity of comprehending tumor behavior and clinical disease, as well as establishing guidelines for monitoring and treating patients affected.

This approach may require a malignant degeneration case to be assessed and staged. The decision on the best treatment should be made by a skin cancer multidisciplinary tumor board including dermatologists, oncologists, pathologists, plastic surgeons, and radiologists [21].

Multidisciplinary tumor boards (MTBs) are meetings that involve different specialists based on the type of cancer, where they discuss all diagnostic and therapeutic issues related to a specific patient before starting therapy [35].

By offering a shared and thoughtful curative program for patients affected by skin cancer, they can overcome potential limitations and biases of therapy determined by one specialist.

NCCN guidelines recommend a multidisciplinary tumor board consultation for complex and high-risk skin cancer cases due to this reason [36,37].

Due to the positive and reliable results of non-surgical therapies for skin cancer, a multidisciplinary approach is increasingly being chosen as the primary and only option in many cases.

Although the standard of care remains tumor excision with clear margins, the reconstructive process may be challenging in cases of locally advanced neoplasms [38]. This may imply that the risk of secondary complications could overweigh the benefits of the surgery, and if the second step cannot be successfully accomplished, maybe the entire procedure should be abandoned in favor of other non-surgical treatments. To ensure patient compliance, expectations, and quality of life, pre-operative planning must take into account all procedures and balance their advantages. In that perspective, the MTB plays a capital role.

A secondary, but not minor, issue concerns the surgeon’s chances of facing legal action for unsatisfactory results [39].

In complex clinical scenarios, surgeons can benefit from the principle of joint responsibility among MTB specialists, as it can prevent them from refusing to operate as a defensive approach.

For all the above-mentioned reasons, surgeons find the MTB to be particularly beneficial when dealing with complex and advanced cases [38].

Given the great variability of clinical presentation [40], with patients developing trichoepitheliomas, cylindromas, spiradenomas, or spiradenocylindromas, an early and accurate clinical diagnosis of suspected malignant degeneration may be arduous. Salient signs of malignant transformations typically consist of rapid growth and bleeding, and lesions located on the trunk seem to show the highest tendency to mutate.

The interaction of proteins such as claudins 3 and 4 and E-cadherin might be involved in neoplasm formation [41,42,43]. Spiradenomas have the potential to transform into aggressive spiradenocarcinomas (SCs) with time, but due to limited reporting of malignant cases, treatment recommendations are made based on case reports and expert opinion [44]. Wargo et al. [45] recently reported a case of a metastatic spiradenocarcinoma that had a high PD-L1 expression even though it did not have estrogen receptor or progesterone receptor expression. Pembrolizumab was given intravenously weekly on the basis of high PD-L1 expression on immunohistochemistry. The patient showed a partial and transient response, thus revealing the possibility of adopting anti-PD-1 immunotherapy as a further therapy in selected cases.

In the literature, local treatment approaches are not standardized. When neoplasms are numerous and/or large, surgery is definitely an option. Portincasa et al. [46] introduced a protocol for tackling spiradenomas of the scalp that involves surgical excision, skin grafting, dermal matrix, expanders, and local flaps. However, for small and few lesions, surgery may not necessarily be the gold standard. Considering other options, such as laser [9], electrocautery, or radiofrequency ablation, may be a viable option. Chaudhary et al. [47] report on a patient who had multiple small papules on his face, and underwent radiofrequency ablation, but did not exhibit any clinical signs of recurrence after a 6-month follow-up period.

In our case, after agreeing with the patient on the necessity of radical excision of the lesion, we discussed the options for reconstruction through local flap or skin grafting. However, the presence of other small lesions in the scalp and the involvement of the underlying periosteum of calvaria below the larger lesion made both approaches unsuitable. Thus, a microsurgical reconstruction with a transplant of the anterolateral thigh flap (ALT) was deemed as the most reliable and comfortable solution in order to achieve free margins of excision and rapid wound healing, allowing the patient to quickly return to his daily activities.

## 5. Conclusions

In conclusion, the evolving landscape of BSS research over the past two decades has illuminated various facets of this complex syndrome. From its molecular underpinnings, to the intricacies of clinical presentation and histopathological features, the literature reflects a collective effort to demystify and manage BSS. As we move forward, collaborative research efforts, multidisciplinary approaches, and an ongoing dialogue between clinicians and researchers will be essential in unraveling the mysteries that still surround Brooke–Spiegler Syndrome.

## 6. Limitations and Future Directions

This paper faces certain limitations inherent to the scope of our research on Brooke–Spiegler syndrome (BSS). First of all, there is a paucity of comprehensive, large-scale studies dedicated to BSS. As a matter of fact, the existing literature predominantly comprises case reports, case series, and observational studies, which have a lower amount of scientific evidence and often lack of comprehensive data on epidemiology, pathogenesis, and treatment.

The collaboration between specialists and researchers is paramount in providing future directions for BSS research. Prospective longitudinal studies are crucial to elucidating the natural history, disease progression, and long-term outcomes of individuals with BSS. Continued efforts in genetic research are essential to unraveling the intricate molecular pathways underlying BSS pathogenesis.

Standardized diagnostic criteria and management guidelines are needed for BSS to ensure consistent and accurate diagnosis, risk stratification, and treatment planning across healthcare settings. Collaborative efforts among multidisciplinary teams are vital for formulating evidence-based guidelines.

## Data Availability

Not applicable.
